# Untargeted metabolomics reveals sex-specific differences in lipid metabolism of adult rats exposed to dexamethasone in utero

**DOI:** 10.1038/s41598-021-99598-x

**Published:** 2021-10-13

**Authors:** Alyssa Murray, Sujeenthar Tharmalingam, Phong Nguyen, T. C. Tai

**Affiliations:** 1 Northern Ontario School of Medicine, Laurentian University, 935 Ramsey Lake Rd, Sudbury, ON P3E 2C6 Canada; 2 Department of Biology, Laurentian University, Sudbury, ON P3E 2C6 Canada; 3 Department of Chemistry and Biochemistry, Laurentian University, Sudbury, ON P3E 2C6 Canada; 4 Biomolecular Sciences Program, Laurentian University, Sudbury, ON P3E 2C6 Canada

**Keywords:** Molecular biology, Endocrine system and metabolic diseases, Biochemistry, Lipids, Metabolomics, Gene expression profiling, Animal disease models, Metabolomics, Disease model, Metabolic diseases, Metabolomics

## Abstract

Prenatal stress through glucocorticoid (GC) exposure leads to an increased risk of developing diseases such as cardiovascular disease, metabolic syndrome and hypertension in adulthood. We have previously shown that administration of the synthetic glucocorticoid, dexamethasone (Dex), to pregnant Wistar–Kyoto dams produces offspring with elevated blood pressures and disrupted circadian rhythm signaling. Given the link between stress, circadian rhythms and metabolism, we performed an untargeted metabolomic screen on the livers of offspring to assess potential changes induced by prenatal Dex exposure. This metabolomic analysis highlighted 18 significantly dysregulated metabolites in females and 12 in males. Pathway analysis using MetaboAnalyst 4.0 highlighted key pathway-level metabolic differences: glycerophospholipid metabolism, purine metabolism and glutathione metabolism. Gene expression analysis revealed significant upregulation of several lipid metabolism genes in females while males showed no dysregulation. Triglyceride concentrations were also found to be significantly elevated in female offspring exposed to Dex in utero, which may contribute to lipid metabolism activation. This study is the first to conduct an untargeted metabolic profile of liver from GC exposed offspring. Corroborating metabolic, gene expression and lipid profiling results demonstrates significant sex-specific lipid metabolic differences underlying the programming of hepatic metabolism.

## Introduction

The long-term effects following fetal exposure to an unfavorable environment poses a great deal of pressure on pregnant women to take care of themselves and follow guidelines such as the consumption of various supplements, avoidance of alcohol or smoking and overall healthy maintenance^1^. The Barker hypothesis states that under situations of maternal stress, many fetal organ systems and functions can undergo ‘programming’ in utero and that this is crucial in developing physiological and metabolic responses into adult life; a phenomenon known as fetal programming^2,3^. There are many types of prenatal stressors such as maternal undernutrition, the dysregulation of hormones, hypoxia, fetal exposure to alcohol or high levels of glucocorticoids (GCs) that can induce a negative programming and result in structural, physiologic, metabolic and epigenetic changes in the offspring^4–7^. Resilience to stressors is decreased and vulnerability is increased by fetal experiences that lead to a biological embedding of response to stressful life events. This vulnerability leads to an increased risk of developing diseases later in life such as cardiovascular disease, metabolic syndrome, insulin resistance and hypertension^4,7–9^.

We have previously shown that the administration of a synthetic GC, dexamethasone (Dex), to pregnant WKY rats produces offspring which exhibit elevated systolic, diastolic and mean arterial blood pressure at adulthood^4,5^. GCs are typically labelled as stress hormones and under situations of stress GCs are released through the activation of the hypothalamic–pituitary–adrenal (HPA) axis, which then coordinates the physiological response to stress via neuroendocrine mechanisms^10^. Glucocorticoid receptors (GRs), which interact with GCs, are expressed in fetal tissues from mid-gestation onwards and in the placenta, and are important in fetal development^10^. Given that they can readily cross the placenta, elevated levels of GCs can result in a stressful in utero environment^11,12^. Clinically, injections of the synthetic GC, Dex, are given to mothers at high risk of preterm labour to accelerate the maturation of fetal lung tissue, decrease the likelihood of respiratory distress syndrome and improve survival^13^. Exogenous GC administration can lead to increased plasma corticosterone levels and prematurely induce significant maturational events^11^. The severity of the programming is often dependent on the dose and timing of the GC exposure^10^.

Through whole transcriptome analysis of the adrenal glands from male offspring exposed to Dex in utero, we reported a disruption of circadian rhythm signaling^14^. There is a significant link between circadian rhythms and metabolism as demonstrated by chronic shift workers, frequent travellers^15,16^ as well as animals subjected to chronodisruption^17,18^. Circadian disruption is strongly associated with metabolic imbalance and an increased risk of metabolic syndrome; which currently describes a group of disorders that often co-occur such as central obesity, insulin resistance dyslipidemia, hyperglycemia and hypertension^19,20^. Factors such as nutrient-lacking high-energy foods along with decreased energy expenditure and a sedentary life-style contribute to metabolic disorders^21^. It has been shown, however, that environmental circadian disruption and genetic aberrant variants in circadian machinery can lead to metabolic disorders^22^. The body is remarkable in its ability to maintain its energy equilibrium but when energy intake exceeds energy expenditure, metabolic fuel is stored as liver glycogen, muscle protein and adipose tissue^19^.

The Dex model of fetal programming is also known as a stress model. Stress is defined as a state of threat to homeostasis or disharmony within the body which results in a variety of physiologic and behavioural responses, that attempt to restore balance within the body^23^. With regard to metabolism, the stress response adapts to the threatening situation by mobilizing the body’s energy stores^24^. There are two categories of stress; acute and chronic. Acute stress has been shown to favour an anorexigenic response where food intake and body weight are reduced in a manner directly proportional to the stress severity^25^. When stressors are prolonged over a longer period of time, it is considered to be chronic and this type of stress leads to increased problems. In a chronic state of stress, the body tries to adapt; which in the short term is beneficial, however, over time this leads to maladaptive changes to physiological responses in addition to the harm of the persistent stressor^23^. While chronic stress initially results in a body weight reduction similar to the acute condition, eventually weight gain occurs as the body makes metabolic adaptations to increase calorie efficiency^24^.

Chronic stress has been associated with perturbations in metabolic homeostasis, which can contribute to the presentation of visceral obesity, type 2 diabetes, atherosclerosis and metabolic syndrome^23^. Rising levels of stress in modern society are correlated with an increasing rate of obesity and metabolic syndrome; both of which have reached levels of epidemic proportion. Stress and these pathologies are interrelated and feed off each other. Chronic HPA axis activation favours visceral fat accumulation, which in turn causes increased stress on the body eventually leading to HPA axis dysfunction^23^. The liver has a central role in all metabolic process of the body and liver energy metabolism is tightly controlled^26^. Dysfunction of liver signaling and metabolic processes can result in metabolic damage and a predisposition to diseases such as type 2 diabetes^26^. Evidence of hepatic programming has been reported as a result of an unfavourable prenatal environment, however, most studies have focused on glucose metabolism^27^.

In this study, we analyzed the liver from adult offspring that were exposed to elevated levels of GCs in utero to identify any metabolic dysregulation that could be associated with the impaired circadian rhythms previously identified in these animals^14^, with the hypertensive phenotype that is present^4,5^, as well as other long-term effects that result from stress in utero. Given what is known of the relationship between impaired circadian rhythms, stress and metabolism, we hypothesized that our fetal programming stress model would result in metabolic impairment of the offspring. Using an untargeted metabolomic approach, this study identified key metabolic pathways impacted as a result of an adverse in utero environment and highlighted significant sex-specific differences in the programming of offspring metabolism.

## Methods

### Animals and tissue collection

WKY rats were purchased from Charles River Laboratory (Montreal, QC, Canada) and housed at Laurentian University’s animal care facility. Protocols regarding animal handling from delivery to tissue collection were previously described^5,14^. All animal protocols were approved by the Laurentian University Animal Care Committee in accordance with guidelines from the Canadian Council on Animal Care and the experiment is in accordance with ARRIVE guidelines. Rats were exposed to a 12-h light–dark cycle and food and water provided ad libitum. Dex-induced fetal programming of WKY rats was previously described^4,5^. Briefly, pregnant WKY females were administered subcutaneous injections of 100 μg/kg/day Dex in 0.9% NaCl with 4% ethanol or saline solution control throughout their third trimester (days 15–21). Resulting offspring were weaned at 3 weeks and housed at 2–3 rats per cage keeping sex consistent. At 19 weeks of age, male and female rats were anesthetized through intraperitoneal injection of 75 mg ketamine and 5 mg xylazine per kg of body weight. Blinding and randomization of animals was completed in this study. Livers and plasma of each sex were collected from 19 week old WKY rats exposed in utero to 100 μg/kg/day Dex and saline (control), frozen on dry ice and stored at − 80 °C until further use. Anesthetizations and subsequent collection of tissues occurred between 10 and 11 am.

### RNA extraction and cDNA synthesis

Total RNA was extracted from the liver using TRI Reagent (Sigma) according to manufacturer’s instructions. Briefly, 50 mg of liver tissue was placed in a microcentrifuge tube with 1 mL TRI reagent along with a stainless steel bead and homogenized using a Tissuelyser for 2 cycles at 30 Hz for 2 min. Homogenized supernatant was mixed with 200 μL of chloroform and centrifuged at 12,000×*g* for 20 min at 4 °C. The aqueous phase containing the RNA was transferred to a fresh tube and mixed with 250 μL of isopropanol before centrifuging at 12,000×*g* for 8 min at 4 °C. The supernatant was discarded and 1 mL of a 70% ethanol solution was used to resuspend the pellet before centrifuging at 7,500×*g* for 5 min at 4 °C. The supernatant was discarded and the pellet air dried. The RNA pellet was then resuspended in DEPC treated water and placed on a thermomixer for 10 min at 37 °C. RNA samples were analyzed using the NanoDrop One spectrophotometer measuring the absorbance ratios at 260/280 nm and 260/230 nm in order to assess concentration and relative purity of samples. Genomic DNA contamination was removed from RNA samples using the DNAseI kit (Sigma) as per manufacturer’s instructions. The DNAse treated RNA samples were reverse transcribed using random primers, mixed dNTPs and Mu-MLV RT (Promega) according to the manufacturer’s instructions.

### Reverse transcribed quantitative PCR (RT-qPCR)

All primers were designed using the primer-BLAST tool (NCBI) to ensure specificity of amplification for target genes. Forward and reverse primer pair sequences for genes of interest were selected, designed and validated using the method previously described^14^. The complete list of validated primer sequences and corresponding information can be found in Supplementary Table S1. RT-qPCR analysis was completed using the QuantStudio 5 Real Time PCR machine (ThermoFisher Scientific) in 15 μL reaction volumes. Each reaction contained 6 ng of the cDNA template, 600 nM of each forward and reverse primer and 7.5 μL of 2X SYBR Green MasterMix (ThermoFisher Scientific). All samples were normalized to housekeeping genes GAPDH and RPL32. The relative fold-change of mRNA transcript level for each gene was calculated according to the ΔΔC_t_ method^28^. Average ΔΔC_t_ and SEM was calculated for each gene.

### Metabolite extraction

Metabolites were extracted from the liver by adding 1 mL of cold metabolite extraction buffer (0.1 M formic acid in methanol: acetonitrile: water [40:40:20]) to a microcentrifuge tube along with 50 mg of liver tissue and a stainless steel bead. Samples were placed in the Tissuelyser for 2–3 cycles of 1 min at 30 Hz with care taken not to heat the samples. Once homogenized, samples were placed at − 20 °C for 1 h and then centrifuged at 16,100×*g* for 5 min at 4 °C. The supernatant was transferred to a fresh tube and stored at − 20 °C while the pellet was resuspended in the extraction buffer. The freezing and centrifuging steps were repeated for a total of three extractions. The final tube containing the supernatant from all three extractions was neutralized with concentrated ammonium hydroxide before being concentrated under vacuum without heat.

### Untargeted metabolomics

Ultra-high performance liquid chromatography (UHPLC) was performed on an UltiMate 3000 UHPLC system (ThermoFisher Scientific) and coupled to a Q Exactive Hybrid Quadrupole-Orbitrap Mass Spectrometer (ThermoFisher Scientific) at BioZone Mass Spectrometry facility (University of Toronto, Toronto, Ontario) as per their standard protocol^29^. Chromatographic separation was achieved using a Hypersil Gold C_18_ column (50 mm × 2.1 mm × 1.9 μm) maintained at a temperature of 40 °C. Chromatographic separation was prepared using a gradient method with a flow rate of 300 μL/min consisting of an aqueous solvent A that was 5 mM ammonium acetate, pH 6.0 in ddH_2_O, and an organic solvent B that was 5 mM ammonium acetate, pH 6.0 in methanol. The injection volume for each sample was set to 10 μL. Prior to injection the samples were resuspended in water, vortexed to mix and spun at maximum speed in a microcentrifuge. The supernatants were collected and used for analysis. The gradient used for each UHPLC run were 0–1 min, 5% B; 1–7 min, linear gradient to 100% B; 7–10 min, 100% B, 10–11 min, linear gradient to 5% B; 11–15 min, 5% B.

The Q Exactive Mass Spectrometer was operated using the heated-electrospray ionization probe (HESI-II; ThermoFisher Scientific) with the following parameters: sheath gas flow rate, 15; auxiliary gas, 5; spare gas, 2; electrospray voltage, 3.5 kV; capillary temperature, 320 °C; S-Lens RF Level, 50. In full scan MS mode the spectra collected were scanned over a mass/charge (m/z) range of 100–1200 atomic mass units at a resolution of 70,000. The AGC target (the predefined value for the accumulation of ions) was set to 3e^6^, the maximum ion injection time (IT) was set to 100 ms. The full MS scan was run on both positive and negative ion modes. After the full scan was completed the setting was switched to data-dependent-MS2 where the top 5 most intense ions from the full MS scan were selected for further fragmentation. The parameters used for the data dependent-MS2 scan were as follows: a resolution of 17, 500; an AGC value of 1e^5^; a maximum IT of 50 ms; an isolation window of 1.0 m/z; and normalized collision energy (NCE) set to 30 eV.

Quality control: the system was recalibrated for mass accuracy and signal response prior to sample analysis. Water controls were used to verify column performance and background noise of untargeted data. Replicates were used to minimize the potential for false positives. Following software analysis, data was manually curated to ensure entries were not false positives. Analysis: Area under the curve was calculated and normalized for each sample. Then, median values were calculated for each sample group.

### Metabolite identification and pathway analysis

Metabolite identification was performed with the ChemSpider database using the molecular weight and retention time from the mass spectrometry runs. Since an untargeted approach does not use reference standards, a putative identification was made and is presented with a level 3 confidence^30^. Metabolic pathway analysis was performed using the Pathway Analysis feature on MetaboAnalyst 4.0 (Xia Lab, McGill University) by means of the compound list option. Then, highlighted pathways were further investigated on the Kyoto Encyclopedia of Genes and Genomes (KEGG)^31^ database (Release 92.0) to identify target genes for further investigation.

### Triglyceride extraction and assay

Approximately 100 mg of liver tissue was added to a microcentrifuge tube containing 1 mL of 5% NP-40 and a stainless steel bead and homogenized using the Tissuelyser for 2 cycles of 2 min at 20 Hz. After homogenization samples were slowly heated to 90 °C in a water bath for 5 min. Samples were cooled to room temperature and then the heating and cooling was repeated once more before lysates were centrifuged for 2 min at 16,100 × g. The supernatant was transferred to a fresh tube and samples diluted tenfold with ddH_2_O. Triglyceride (TG) concentrations were determined using the Triglyceride Quantification Assay Kit from Abcam (ab65336). TG standards were prepared from a 1 mM standard included in the kit to create a standard curve ranging from 0 to 10 nmol. Samples and standards were added to a clear 96-well assay plate as per manufacturer’s instructions. Briefly, 2 μL of Lipase was added to each well and incubated for 20 min at room temperature. A reaction mix of assay buffer, probe and enzyme mix was prepared as per manufacturer’s instructions and 50 μL added to each well. The plate was incubated at room temperature for 60 min in the dark before being measured using the PowerWave XS spectrophotometer and Gen5 software (Biotek) at 570 nm. This assay quantified the amount of triglycerides present in the livers of offspring by comparing the absorbance of the colorimetric assay to the TG standard curve and then normalizing to the weight of tissue used for the analysis. Plasma was assayed directly.

### Statistics

All statistical analyses were performed using GraphPad Prism software (La Jolla, CA, USA). All data are presented as mean ± SEM (n = 8). G power analysis: a cut-off of twofold difference to determine biological relevance was used to determine that an n of 8 was appropriate for this study. Interquartile range (IQR) was calculated for all treatment groups and outliers were determined when measurements were lower than the first quartile or higher than the third quartile by magnitudes greater than 1.5 * IQR. Statistical significance between two groups (i.e. Saline and Dex animals) was determined by an unpaired t-test. Statistical significance between greater than two treatment groups was determined by one-way ANOVA followed by post-hoc Tukey’s multiple comparison test. Values of p ≤ 0.05 were considered statistically significant.

## Results

### Untargeted metabolomics screen

A liquid chromatography tandem mass spectrometry based untargeted metabolomic screen was performed using the livers of eight male and female adult offspring that were exposed to saline or Dex in utero. A total of 704 compounds were identified during the chromatographic separation and their molecular weight was determined using mass spectrometry. Using the ChemSpider database, 460 of the 704 compounds were positively identified. Metabolite data of prenatal Dex exposed female offspring compared with their saline-treated controls showed 18 significantly dysregulated metabolites (p < 0.05) with 7 also meeting a secondary cut-off (fold change > 2 or < 0.5) (Table 1). The metabolite data from prenatal Dex exposed male offspring compared to their saline-treated controls showed 12 significantly dysregulated metabolites with 8 of those also meeting the secondary fold-change cut-off (Table 2). Further information from the metabolomic results for female and male offspring such as molecular weight and retention time is presented in Supplementary Tables S2 and S3 respectively.

**Table 1:** Significant results from the untargeted metabolomic screen of liver from female Dex exposed offspring compared to their saline controls. Compounds identified as ** indicate metabolites that reached the two cut-off criteria (p < 0.05 and fold change > 2 or < 0.5) while compounds identifed as * indicate metabolites that reach the p < 0.05 criteria only. Upregulations are presented first in descending order with downregulations following (n = 8).

Name	Fold change	p value	Description	Pathway(s)
2,3,4,5-Tetrahydroxypentanal**	6.228	1.31E−07	Heterosaccharide	Upstream of glycerol metabolism
Ophthalmic acid**	4.303	3.55E−04	L-glutamine derivative; analogue of glutathione	Cys and Met metabolism
Propionylcarnitine**	2.059	0.0269	Acylcarnitine; a fatty ester lipid molecule	Lipid/fatty acid (FA) metabolism; lipid transport, oxidation of branched chain FAs
Adenosine*	1.970	0.0047	Component of DNA and RNA; neurotransmitter and potent vasodilator	Purine metabolism; cAMP signaling pathway
Adenine*	1.911	0.0109	Purine nucleobase	Purine metabolism
Creatinine*	1.905	0.0012	Product of creatine phosphate	Arg and Pro metabolism
Spermidine*	1.869	8.44E−05	Polyamine; helps stabilize membranes and nucleic acid structures	Arg, Pro, Ala, and glutathione metabolism; bile secretion
Glutathione disulfide*	1.775	0.0416	Oxidized form of glutathione	Glutathione metabolism
Choline*	1.683	0.0221	Precursor of acetylcholine	Many pathways
L-(−)-methionine*	1.667	0.0493	Essential amino acid	Cys and Met metabolism
Hypoxanthine*	1.634	0.0354	Purine derivative	Purine metabolism
L-(+)-Ergothioneine*	1.558	0.0385	Metabolite of His; antioxidant properties	Histidine metabolism
3-hydroxyisovaleryl carnitine*	1.486	0.0186	Intermediate of FA oxidation	Lipid/FA metabolism; lipid transport
Nicotinamide*	1.464	0.0433	Pyridine derivative; precursor for NAD + /NADH and NADP + /NADPH	Nicotinate/Nicotinamide metabolism
Phosphatidylethanol-amine (18:2/18:2)**	0.006	6.18E-05	Phosphatidylethanol-amine lipid	Glycerophospholipid metabolism; lipid transport
Lactosylceramide (d18:1/12:0)**	0.216	0.0018	Important ceramide; assists in stabilizing plasma membrane	Phospholipid/ sphingolipid and lipid/FA metabolism; lipid transport
Linolenelaidic acid**	0.308	0.0116	Polyunsaturated omega-6 long chain fatty acid	Lipid/FA metabolism; lipid transport
Phosphatidylserine (14:0/14:1)**	0.491	0.0012	Phosphatidylserine lipid	Glycerophospholipid and lipid metabolism; lipid transport

**Table 2:** Significant results from the untargeted metabolomic screen of liver from male Dex exposed offspring compared to their saline controls. Compounds identified as ** indicate metabolites that reached the two cut-off criteria (p < 0.05 and fold change > 2 or < 0.5) while compounds identified as * indicate metabolites that reach the p < 0.05 criteria only. Upregulations are presented first in descending order while downregulations follow (n = 8).

Name	Fold change	p value	Description	Pathway(s)
Vanillin 4-sulfate**	3.004	0.0035	Polyphenol metabolite	
L-Carnitine tetradecanoyl ester**	0.203	0.0329	Carnitine with FA attached; beta oxidation of long chain FAs	Lipid metabolism
Alpha-aminoadipic acid**	0.212	0.0472	Intermediate of metabolism of Lys	Lys biosynthesis and degradation
PC(18:1(9Z)/22:6(4Z,7Z,10Z,13Z,16Z,19Z))**	0.265	5.42E-04	A type of phosphatidylcholine; a glycerophospholipid	Glycerophospholipid/ lipid metabolism
LysoPC(18:3(9Z,12Z,15Z))**	0.355	0.0417	Monoglycerol phospholipid	Phospholipid, lipid and FA metabolism; lipid transport
Eicosapentanoic acid**	0.430	0.0486	Important polyunsaturated fatty acid found in fish oils	Alpha linolenic acid and linoleic acid metabolism; lipid metabolism
Uracil**	0.464	0.0405	Pyrimidine nucleobase	Pyrimidine metabolism; beta-Ala metabolism
DL-Carnitine**	0.487	0.0106	Conditionally essential metabolite; transports fat into mitochondria	Thermogenesis; bile secretion; fatty acid metabolism/β-oxidation
Xanthine*	0.623	0.0124	Intermediate of the degradation of AMP to uric acid	Purine metabolism
DL-Tyrosine*	0.630	0.0113	Essential amino acid; more needed under stress; rapidly metabolized	Cathecholamine biosynthesis; Phe and Tyr metabolism
Hypoxanthine*	0.717	0.0406	Purine derivative; present in anticodon of tRNA	Purine metabolism
DL-Tryptophan*	0.770	0.0291	Essential amino acid; precursor for melatonin and serotonin	Transcription/translation

Across both female (Table 1) and male (Table 2), results show that lipids, fatty acids, carnitine and carnitine intermediates, as well as purine derivatives were common dysregulated metabolites. Even though there were commonalities in metabolites identified between females and males, the only specific metabolite that was dysregulated in both sexes was the purine derivative hypoxanthine. The dysregulation of this compound, however, was not the same in both sexes with females displaying an upregulation while males exhibited a downregulation. With the exception of one metabolite, males displayed a downregulation in all significantly dysregulated metabolites. Females, however, showed that metabolites involved in lipid metabolism, with the exception of carnitine derivatives, were downregulated but all other metabolites showed an upregulation.

### Metabolic pathway analysis

To understand the relevance of the dysregulated metabolites in regard to specific biological processes, a metabolic pathway analysis was performed using the online tool MetaboAnalyst 4.0. For this analysis, all metabolites that were significant (p < 0.05) were utilized. From the pathway analysis there were 12 metabolic pathways highlighted for the females (Supplementary Table S4) and 16 for the males (Supplementary Table S5). The MetaboAnalyst pathway analysis tool revealed that the females had three significantly dysregulated pathways: purine metabolism (match status 3/66 metabolites in the pathway; p = 0.0206), glutathione metabolism (match status 2/28; p = 0.0263) and glycerophospholipid metabolism (match status 2/36; p = 0.0421). The males had significance in four metabolic pathways including glycerophospholipid metabolism (match status 2/36; p = 0.0221), phenylalanine, tyrosine and tryptophan biosynthesis (match status 1/4; p = 0.0263), linoleic acid metabolism (match status 1/5; p = 0.0327) and aminoacyl-tRNA biosynthesis (match status 2/48; p = 0.0379).

### Selection of metabolic pathways and creation of gene panels

Three metabolic pathways were selected to undergo further investigation from the metabolomic screen as they showed the greatest significance and/or were impacted in both sexes. These included glycerophospholipid metabolism, purine metabolism and glutathione metabolism. Metabolites from these pathways which showed dysregulation were located in their metabolic pathway on the KEGG database^31^ and genes/enzymes that appeared up and downstream were selected for gene panels. The glycerophospholipid metabolism gene panel consisted of 20 genes that play an essential role in both phospholipid and general lipid metabolism (Table 3). The purine metabolism gene panel was created with 5 genes pertaining specifically to the purines that were identified from the screen (Table 4). The glutathione metabolism panel was created with 7 genes related to general and specific antioxidant responses (Table 5). We have previously reported circadian rhythm dysregulation in the adrenal glands of male offspring subjected to Dex exposure in utero^14^ and therefore used our previously created circadian rhythm gene panel to compare possible effects in the liver (Table 6).

**Table 3:** Glycerophospholipid general lipid metabolism gene panel RT-qPCR results. mRNA levels in the livers of 19 week old prenatally DEX-exposed offspring relative to saline control. Fold changes in gene expression were calculated by relative quantification (ΔΔCt) of RT-qPCR threshold cycles (Ct) as per Livak and Schmittgen^28^ using mean Ct values of housekeeping genes GAPDH and Rpl32. Data is expressed as mean fold change of Dex relative to the saline group ± SEM (n = 8). Unpaired t-test: Statistical significance between groups is shown by: * p < 0.05, ** p < 0.01, *** p < 0.001.

Gene	Description	Sex	Fold change ± SEM
ACSL1	Acyl-CoA synthetase	Male Female	0.93 ± 0.14 1.24 ± 0.16
CRAT	Carnitine acetyl-transferase	Male Female	0.82 ± 0.13 1.17 ± 0.26
CPT1A	Carnitine palmitoyltransferase 1	Male Female	1.18 ± 0.36 2.54 ± 0.28 ***
CPT2	Carnitine palmitoyltransferase 2	Male Female	0.91 ± 0.14 1.44 ± 0.19 *
ACADL	Acyl-CoA dehydrogenase long chain	Male Female	0.89 ± 0.15 1.21 ± 0.08 *
ACADM	Acyl-CoA dehydrogenase medium chain	Male Female	0.96 ± 0.12 1.10 ± 0.10
ECH1	Enoyl-CoA hydratase	Male Female	1.07 ± 0.14 1.66 ± 0.19 **
HADH	L-hydroxyacyl-CoA dehydrogenase	Male Female	0.98 ± 0.08 1.23 ± 0.12 *
ACAT1	Thiolase	Male Female	0.94 ± 0.15 1.15 ± 0.08 *
PCCA	Propionyl-CoA carboxylase alpha	Male Female	0.92 ± 0.05 1.23 ± 0.12 *
PCCB	Propionyl-CoA carboxylase beta	Male Female	0.99 ± 0.09 1.18 ± 0.08 *
MCEE	Methylmalonyl-CoA racemase	Male Female	1.14 ± 0.12 1.26 ± 0.14 *
MMUT	Methylmalonyl-CoA mutase	Male Female	1.01 ± 0.10 1.30 ± 0.12 **
ECI1	Enoyl-CoA isomerase	Male Female	0.99 ± 0.27 1.21 ± 0.27
DECR1	2,4-dienoyl-CoA reductase	Male Female	1.00 ± 0.19 1.27 ± 0.14 *
PLA1A	Phospholipase A1	Male Female	0.97 ± 0.16 1.41 ± 0.18 *
PLA2G2A	Phospholipase A2	Male Female	1.05 ± 0.07 1.21 ± 0.16
PLCG1	Phospholipase C	Male Female	1.10 ± 0.14 1.11 ± 0.12
PLD1	Phospholipase D	Male Female	1.09 ± 0.34 1.26 ± 0.34
GDE1	Glycerophosphodiester phosphodiesterase	Male Female	0.96 ± 0.15 1.45 ± 0.16 **
PEMT	Phosphatidylethanolamine N-methyltransferase	Male Female	1.02 ± 0.11 0.97 ± 0.16
PPARA	Peroxisome proliferator-activated receptor alpha	Male Female	0.87 ± 0.31 1.31 ± 0.33

**Table 4:** Purine metabolism gene panel RT-qPCR results. mRNA levels in the livers of 19 week old prenatally Dex-exposed offspring relative to saline control. Fold changes in gene expression were calculated by relative quantification (ΔΔCt) of RT-qPCR threshold cycles (Ct) as per Livak and Schmittgen^28^ using mean Ct values of housekeeping genes GAPDH and Rpl32. Data is expressed as mean fold change of Dex relative to the saline group ± SEM (n = 8). Unpaired t-test: Statistical significance between groups is shown by: * p < 0.05, ** p < 0.01.

Gene	Description	Sex	Fold change ± SEM
NT5C2	5′-nucleotidase	Male Female	0.96 ± 0.13 1.19 ± 0.12
ADA	Adenosine deaminase	Male Female	0.76 ± 0.11 ** 0.79 ± 0.11 **
PNP	Purine nucleoside phosphorylase	Male Female	1.02 ± 0.08 1.08 ± 0.10
XDH	Xanthine oxidase/dehydrogenase	Male Female	1.18 ± 0.09 * 1.09 ± 0.13
UOX	Uricase	Male Female	1.00 ± 0.09 0.89 ± 0.05 *

**Table 5:** Glutathione metabolism gene panel RT-qPCR results. mRNA levels in the livers of 19 week old prenatally Dex-exposed offspring relative to saline control. Fold changes in gene expression were calculated by relative quantification (ΔΔCt) of RT-qPCR threshold cycles (Ct) as per Livak and Schmittgen^28^ using mean Ct values of housekeeping genes GAPDH and Rpl32. Data is expressed as mean fold change of Dex relative to the saline group ± SEM (n = 8). Unpaired t-test: Statistical significance between groups is shown by: * p < 0.05.

Gene	Description	Sex	Fold change ± SEM
GSR	Glutathione reductase	Male Female	1.19 ± 0.30 1.40 ± 0.22 *
GPX1	Glutathione Peroxidase	Male Female	0.98 ± 0.21 0.97 ± 0.18
GGT1	Glutathione hydrolase	Male Female	1.25 ± 0.18 0.63 ± 0.31
SMS	Spermine synthase	Male Female	0.96 ± 0.10 1.02 ± 0.12
CAT	Catalase	Male Female	0.99 ± 0.07 1.08 ± 0.08
SOD1	Superoxide dismutase 1	Male Female	1.05 ± 0.23 0.73 ± 0.20 *
SOD2	Superoxide dismutase 2	Male Female	1.05 ± 0.18 0.84 ± 0.14

**Table 6:** Circadian rhythm gene panel RT-qPCR results. mRNA levels in the livers of 19 week old prenatally Dex-exposed offspring relative to saline control. Fold changes in gene expression were calculated by relative quantification (ΔΔCt) of RT-qPCR threshold cycles (Ct) as per Livak and Schmittgen^28^ using mean Ct values of housekeeping genes GAPDH and Rpl32. Data is expressed as mean fold change of Dex relative to the saline group ± SEM (n = 8). Unpaired t-test: Statistical significance between groups is shown by: * p < 0.05.

Gene	Description	Sex	Fold change ± SEM
CLOCK	Clock circadian regulator	Male Female	0.88 ± 0.10 0.94 ± 0.19
BMAL1	Brain and muscle ARNT like protein 1	Male Female	0.65 ± 0.42 0.66 ± 0.65
NPAS2	Neuronal domain PAS protein 2	Male Female	0.75 ± 0.49 0.83 ± 0.56
PER1	Period circadian protein homolog 1	Male Female	0.95 ± 0.38 1.59 ± 0.43
PER2	Period circadian protein homolog 2	Male Female	1.56 ± 0.21 * 0.93 ± 0.51
PER3	Period circadian protein homolog 3	Male Female	1.28 ± 0.79 1.23 ± 1.00
CRY1	Cryptochrome circadian clock 1	Male Female	0.83 ± 0.46 1.26 ± 0.26
CRY2	Cryptochrome circadian clock 2	Male Female	1.09 ± 0.15 1.12 ± 0.23
REV-ERBA	Nuclear Receptor Subfamily 1 Group D Member 1	Male Female	0.70 ± 1.05 0.66 ± 0.78
METTL3	N6-adenosine-methyltransferase 70 kDa subunit	Male Female	1.10 ± 0.14 0.80 ± 0.17
FBXL3	F-box and leucine rich repeat protein 3	Male Female	1.04 ± 0.16 0.92 ± 0.19
CSNK1D	Casein kinase I isoform delta	Male Female	1.01 ± 0.10 0.92 ± 0.11
CSNK1E	Casein kinase I isoform epsilon	Male Female	0.78 ± 0.20 1.12 ± 0.44

### RT-qPCR analysis of gene panels

In order to better understand the results from the metabolomic screen, gene panels from top dysregulated metabolic pathways were analyzed using RT-qPCR. Across all four gene panels analyzed for glycerophospholipid and general lipid metabolism (Table 3), purine metabolism (Table 4), glutathione metabolism (Table 5) and circadian rhythms (Table 6), females had 17 significantly dysregulated genes in adult offspring prenatally exposed to Dex in utero compared to their saline counterparts. More specifically, 11/15 lipid metabolism genes, 2/6 phospholipid metabolism genes, 2/5 purine metabolism genes, 2/7 glutathione metabolism genes and 0/14 circadian rhythm signaling genes were affected. All 11 dysregulated genes in the lipid metabolism pathway (*CPT1A, CPT2, ACADL, ECH1, HADH, ACAT1, PCCA, PCCB, MCEE, MMUT* and *DECR1*) and the 2 dysregulated genes in the phospholipid metabolism pathway (*PLA1A* and *GDE1*) showed an upregulation, which indicates an overall activation of lipid and phospholipid metabolism in adult female livers exposed to GC in utero. The two significantly dysregulated purine metabolism genes (*ADA* and *UOX*) both showed a downregulation. Finally, of the two significantly dysregulated glutathione metabolism genes *GSR* was upregulated while *SOD1* was downregulated. In contrast, male offspring exposed to GC in utero showed a different pattern with only 3 significantly dysregulated genes compared to their saline controls across the four gene panels. The two significantly dysregulated purine metabolism genes were *ADA*, which was downregulated and *XDH*, which was upregulated. The significantly dysregulated gene in the circadian rhythm signaling panel was *PER2*, which showed an upregulation.

### Triglyceride (TG) assay

**Figure 1:**
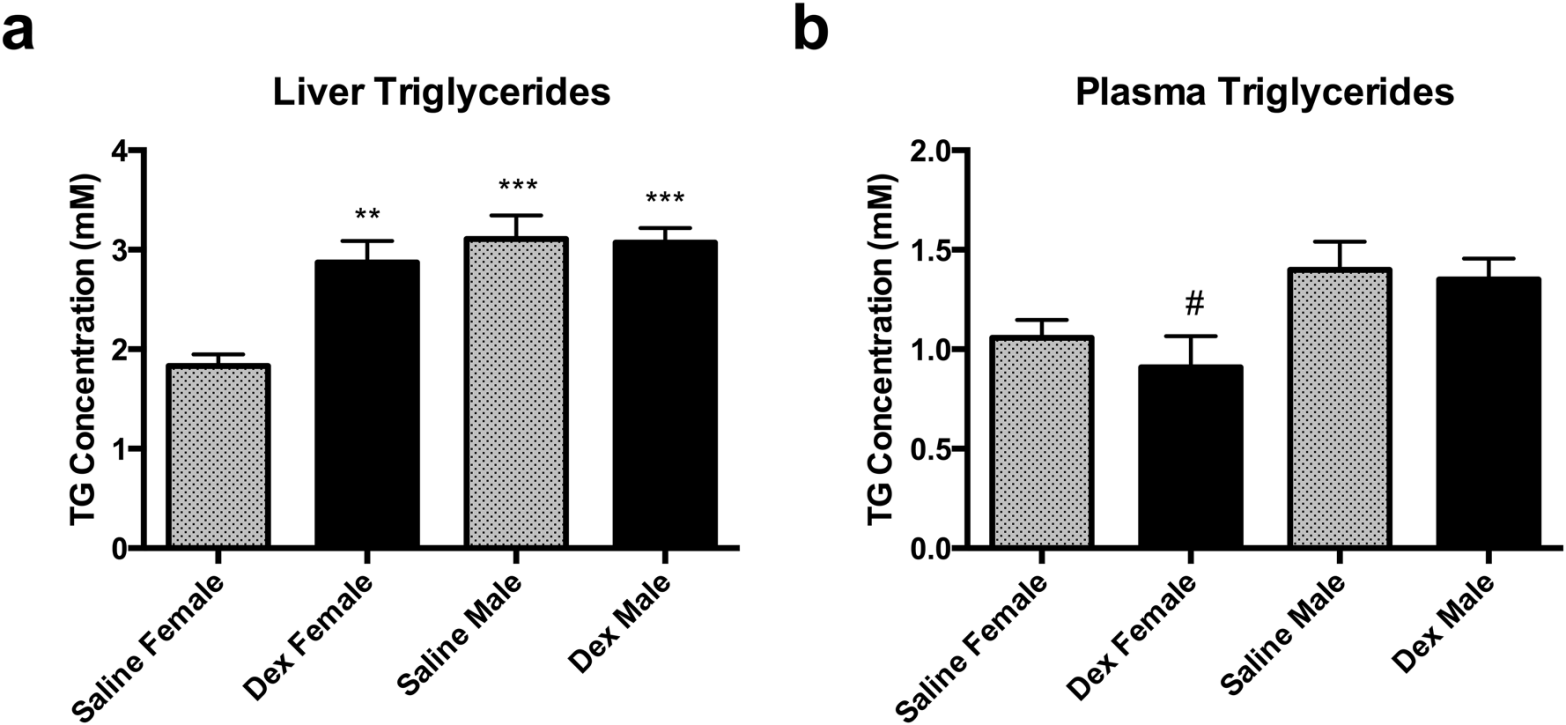
Triglyceride concentrations in the liver and plasma of saline and Dex exposed offspring. Levels of triglycerides were measured in the liver (**a**) and plasma (**b**) by the Triglyceride Quantification Assay (Abcam) in a colorimetric assay at 570 nm. For the livers, the triglyceride concentration was normalized to the weight of the tissue used for the analysis. The plasma samples were assay directly. The triglyceride concentrations in mM are represented graphically. Statistical significance between saline and Dex groups and sex was determined by one-way ANOVA with a post-hoc Tukey test. Data are presented as mean ± SEM (n = 8). Statistical significance between groups is shown by: * p < 0.05, ** p < 0.01, *** p < 0.001. The * indicates significance when compared to saline female group and the # indicates significance when compared to the saline male group.

Since lipid metabolism was strongly implicated in the metabolomic screen and especially dysregulated in the female gene expression analysis, a triglyceride assay was performed on both liver tissue and plasma of the Dex-exposed offspring and their saline controls. In the liver, adult females exposed to Dex in utero had elevated TG content (2.87 ± 0.21 mM) compared to saline control (1.83 ± 0.12 mM) (Fig. 1a). In contrast, the TG content in the liver of adult males exposed to Dex in utero (3.07 ± 0.15 mM) was similar to saline (3.11 ± 0.24 mM); however were both higher compared to the saline female liver. Compared to the liver, TG content in the plasma showed no effect from in utero Dex exposure in both males (1.35 ± 0.10 mM) and females (0.91 ± 0.16 mM) compared to saline control (male: 1.40 ± 0.14 mM; female: 1.06 ± 0.09 mM) (Fig. 1b); however note that in general, plasma TG levels are higher in males compared to females. The plasma samples also had much lower levels of TGs, hovering around 1 mM compared to the liver, which showed 2–3 mM TG concentrations.

## Discussion

Given the strong link between circadian rhythm and metabolism^19^, the impact the prenatal environment has on the development of cardio-metabolic disorders^2,32^ and the monumental role of the liver in metabolism, we wanted to examine if prenatal Dex exposure results in changes to postnatal adult liver metabolism. This study is the first to produce a metabolic profile of offspring exposed to Dex in utero and identified lipid metabolism to be significantly impacted in a sex-specific manner. Comparing gene expression results to their physiological endpoints shown through metabolites, we have compiled strong evidence suggesting an activation of lipid metabolism in female offspring. Through quantifying triglyceride (TG) content, we have also identified a significant increase in the amount of TGs present in the liver of female offspring, which may be playing a factor in the lipid metabolism activation.

From the gene expression results in particular, it was clear that females were more metabolically affected than the males. Females displayed 17 dysregulated genes with the greatest amount of dysregulation seen in the lipid metabolism panel with 11 genes affected, and the phospholipid metabolism panel with 2 genes affected. Interestingly, all dysregulated genes in lipid and phospholipid metabolism were upregulated. Carnitine palmitoyltransferase 1 (*CPT1A*) showed the largest upregulation; with *CPT2* also upregulated, albeit not as strongly. *CPT1A* and *CPT2* are involved in the formation of acylcarnitines to allow for transport of lipids from the cytosol to the mitochondria and the product of this reaction is usually palmitoylcarnitine^33^. In the female metabolomics it was seen that propionylcarnitine, an acylcarnitine, was increased, and an intermediate of fatty acid oxidation, 3-hydroxy-isovalerylcarnitine was also upregulated. The dysregulation in these metabolites match with the increase in *CPT1A* and *CPT2* gene expression since these carnitine intermediates are involved in the metabolic reaction but not consumed.

The remaining 9 dysregulated genes in the lipid metabolism panel participate in fatty acid β-oxidation and in the female metabolomics, the following lipids were found to be dysregulated: phosphatidylethanolamine, phosphatidylserine, lactosylceramide, and linolenelaidic acid (Table 1). The widespread upregulation of β-oxidation enzymes indicates that an increased metabolism of lipids is occurring and matches the decreased lipid levels (Table 3). The two dysregulated genes involved in phospholipid metabolism were *PLA1A*, which hydrolyzes fatty acids at the sn-1 position of phosphatidylserine^34^ and *GDE1*, which hydrolyzes glycerophospholipids in general^35^. In the metabolomics results, a phosphatidylethanolamine and phosphatidylserine were each shown to be downregulated and similarly to the lipid metabolism discussed above, this upregulation in gene expression matches the decreased levels of phospholipids.

Within lipid metabolism there are also several rate-limiting steps and/or inhibitors that could impact the rate of metabolism. For instance, CPT1 activity, discussed earlier, is known to be inhibited by the metabolite malonyl-CoA^36^. Insulin and glucose have been found to increase the concentration of malonyl-CoA while exercise and glucagon signaling lead to the inactivation of the acetyl carboxylase enzymes (ACC1/2), which produce malonyl-CoA^36^. Since GC-exposed offspring have an increased risk of metabolic syndrome, which is often accompanied by high levels of glucose and insulin resistance, it would be interesting to investigate a potential link between CPT1 activity and disease phenotype. Perhaps the increased gene expression in *CPT1A* is a programmed metabolic adaptation to overcome CPT1 inhibition and combat metabolic disease. The levels of *CPT1A* have also been shown to oscillate and there is evidence indicating that this oscillation is dependent on the PER proteins^37^. Since we know that there is circadian disruption in these animals^14^, this may be playing a role in CPT1 activation. It is also interesting to note that estrogen supplementation in rats showed an increase in CPT1 enzyme activity and perhaps this is contributing to the sex difference seen in *CPT1A* expression as a result of fetal programming^38^.

Females also exhibited 2 genes dysregulated in purine metabolism; *ADA*, which converts AMP to adenosine, and *UOX*, which converts uric acid to allantoin^39^. In the female metabolomics it was seen that both adenosine and adenine were upregulated. The downregulation in *ADA* indicates a decreased rate of metabolism of purine intermediates. The metabolomics also indicated that hypoxanthine was upregulated and since hypoxanthine is an upstream metabolite of the UOX reaction, this gives greater confidence that purine metabolism is decreased.

Lastly, the females had 2 genes dysregulated in the glutathione metabolism pathway. *GSR*, the enzyme responsible for reducing oxidized glutathione disulfide to glutathione (an important cellular antioxidant^40^), was upregulated. This matches the metabolite data where females showed an upregulation in glutathione disulfide. This upregulation in *GSR* could be reacting to an increased amount of reactive oxygen species (ROS) within the liver (a phenotype seen in the heart and kidneys of programmed offspring^41^) in order to regenerate the antioxidant activity of glutathione. *SOD1* was also affected in this pathway with a downregulation. SOD1 is the cytoplasmic variant of superoxide dismutase, SOD2 is the mitochondrial form and SOD3 an extracellular form, but all share a similar role of destroying superoxide radicals and providing an antioxidant response^42^. This investigation examined both SOD1 and SOD2 and showed that there is a decrease in cytoplasmic antioxidant expression compared to the mitochondrial in Dex exposed female offspring. We have previously shown that the transcription of SOD1 is particularly susceptible to changes in methylation status in female Dex-exposed offspring^6^. Therefore, it is possible that the decrease in SOD1 expression is the product of a programmed hypermethylation in female offspring of prenatal stress.

Males showed no dysregulation in lipid metabolism or phospholipid metabolism, which was surprising given 5/7 of the most significantly dysregulated metabolites were involved in lipid metabolism, and glycerophospholipid metabolism was the top dysregulated pathway seen in the male pathway analysis. It is possible that the Dex exposed males have adapted to become more metabolically efficient than their saline controls and are able metabolize a greater number of lipid molecules while maintaining the same level of transcription and translation. This is actually indicative of what occurs to chronically stressed individuals^23,24^. When the body is in a persistent state of stress it tries to adapt and in regard to metabolism it adapts to increase calorie efficiency^24^.

Males exhibited dysregulation in purine metabolism with *ADA* downregulated and *XDH* upregulated. With both enzymes in the same pathway it is curious they exhibit opposite effects. In the male metabolomics it was seen that xanthine and hypoxanthine were slightly downregulated. Perhaps the upregulation seen in *XDH* expression indicates increased metabolism, which would result in decreased metabolites. Xanthine oxidase/dehydrogenase (XDH) activity, which catalyzes the reaction of hypoxanthine to xanthine, and finally to uric acid generates large amounts of superoxide anions which can cause hepatic damage and inflammation through ROS^43^. For *ADA*, neither AMP or adenosine were dysregulated in the metabolomic screen but since xanthine is an intermediate of the degradation of AMP to uric acid this downregulation in ADA may indicate the pathway is concentrating toward the end product of uric acid. High levels of uric acid, also known as hyperuricemia are implicated in the initiation and progression of many manifestations of metabolic syndrome including hypertension^44^. It has been suggested that uric acid can penetrate vascular smooth muscle fibres and results in a rise of arterial pressure, vascular smooth muscle cell hypertrophy and hypertension^45^. It is also interesting to note that in our previous transcriptome study of the adrenal glands of male offspring, purine metabolism was a top signaling pathway that was dysregulated^14^.

The last gene dysregulated in the males was the circadian rhythm gene *PER2*. This was the only circadian gene significantly upregulated in either sex. Interestingly, PER2 is a circadian clock protein that has been shown to have intracellular roles apart from the core circadian oscillator in the liver^19,46^. PER2 can bind to the nuclear receptors PPARα, PPARγ and Rev-Erbα and plays a role in controlling white adipose and liver tissue metabolism^47,48^. Further analysis of both PPARα and Rev-Erbα showed no changes in gene expression; however, given that PER2 binds to these receptors and has not been shown to actively influence transcription, it is possible that PER2 is acting with these receptors in the liver to help control metabolic perturbations caused by fetal programming.

Due to the lack of circadian rhythm disruption in the liver, it appears as though the hepatic circadian clock was not affected during the programming, however, metabolism was. It is known that the circadian clock can be autonomous and has intrinsic clock function even in the absence of functional clocks in all other tissues^49^. While external inputs are needed to have a fully functional circadian control over metabolites, the liver circadian clock retains autonomy restricted to specific genes and metabolic pathways, mostly within carbohydrate metabolism^49^. The liver seems to be resilient with regard to nutritional stressors as when placed on a high fat diet, the liver showed high temporal metabolite correlation to a chow diet compared to serum or brown adipose tissue that had essentially lost metabolite correlations^50^. Given that the Dex exposed offspring have been shown to exhibit altered circadian rhythms in the adrenal glands, it is possible this is playing a role in their dysregulated metabolism possibly through ill-timed GC release by the adrenal glands^14^, or by mistimed signals from brain areas, such as the hypothalamus, which drive feeding responses.

As lipids and their metabolism were strongly implicated in this investigation, the concentration of triglycerides (TGs) was determined in both the liver and plasma of Dex-exposed offspring and revealed that the amount of TGs in the liver of Dex females is significantly upregulated compared to their saline counterparts. Also interesting is that the concentration of TGs in Dex females match TG concentrations in the males. Again, results show that females are more affected than the males; something that is not common in fetal programming^51^. It is possible that this dysregulation in females is serving a potentially protective role. The increased concentration of liver TGs could be driving the activation of lipid metabolism seen through the gene expression data (Table 3). This increase in metabolism through increased fatty acid β-oxidation and activation of mitochondria (the site of the metabolism) is likely driving the increase in glutathione metabolism and its subsequent antioxidant effects as tissues with increased lipid metabolism are at greater risk of oxidative stress^52^.

The robust effect on lipid metabolism in females compared to males may help to explain sex discrepancy in the fetal programming disease progression. There are many sex differences documented with regard to lipid metabolism as well as antioxidant response. Females generally exhibit more beneficial metabolic profiles than males, which are linked to both sex hormones as well as effect of sex chromosomes^53,54^. Disease risk for diabetes, non-alcoholic fatty liver disease (NAFLD) and hypertension are all higher in men than women until post menopause, which suggests hormone regulation^53^. Women have also been shown to have differences in lipid metabolism where they burn fat more preferentially during exercise compared to men, however, are shown to revert to a state of reduced fatty acid oxidation (FAO) immediately afterward^38,55^. The overall greater fat mass in women has been attributed to a greater efficiency in conserving energy and storing fat and it has been indicated that estrogen may have an inhibitory effect on FAO in the liver^55^. With such prominent differences in lipid metabolism between sexes, it is not surprising we saw distinct metabolic profiles following prenatal stress.

With regard to antioxidant response, in vivo biomarkers of young men and women show higher oxidative stress in males while females show a greater antioxidant potential^56^, which may explain the differences in antioxidant response seen between male and female offspring. It is also known that excessive activation of GR signaling antagonizes NRF2-mediated cytoprotection from oxidative stress through their interaction regardless of the presence of GREs^57^. We have previously shown increased mRNA levels of GR in the adrenal gland of Dex-exposed offspring^4,5^, which would result in decreased NRF2 binding to antioxidant response elements and a higher level of oxidative stress. It is possible that GR signaling is equally dysregulated in the livers of these animals, which is contributing to the phenotype seen.

It has been shown that programmed hyperlipidemia and hypertension can be mediated by a postnatal diet supplemented with ω-3 fatty acids^58^. Supplementation with carnitine resists blood pressure elevation in hypoglycemic animals, keeping it within normal range, while carnitine deficiency lead to hypoglycemia-induced hypertension^59^. Carnitine has also been shown to have effects on weight loss and body composition^60^. In this study we found that female offspring had increased levels of carnitine and its derivatives, while males showed lowered levels of carnitine. It is possible this discrepancy in carnitine content is contributing to the sex specific differences in the hypertensive phenotype of the fetal programming model^4,5^. More investigation will be needed to determine if these supplements or other diets could be beneficial in reducing the harm of prenatal stress seen in adult offspring.

In conclusion, this study provides evidence of programming of liver metabolism as reflected by permanent changes in the metabolic pathways investigated. We determined metabolic profiles in the livers of GC exposed offspring where sex-specific effects as the result of fetal programming was observed. Females displayed increased lipid and glutathione metabolism, increased levels of liver TGs and decreased purine metabolism, while males showed increased purine metabolism and a hypothesized increase in lipid metabolism efficiency. This study adds further evidence to the idea that hepatic metabolism can be programmed by events during early life and that these programming events are sex-specific.

## Supplementary Information

Supplementary Information.
