# Supplementary material for: Untargeted metabolomics reveals sex-specific differences in lipid metabolism of adult rats exposed to dexamethasone in utero
Source: Sci Rep. 2021 Oct 13;11:20342. doi: 10.1038/s41598-021-99598-x (PMC8514544; doi:10.1038/s41598-021-99598-x)
Supplement: Supplementary file 1 — Supplementary Information. [file 41598_2021_99598_MOESM1_ESM.docx]

**Supplementary Tables**

**Supplementary Table S1.** Primer sequences used for reverse transcription quantitative PCR (RT-qPCR) with amplicon sizes and annealing temperatures

| **Gene/**  **Accession ID** | **Sequence (5’-3’)** | **Amplicon Size (bp)** | **Annealing Temp (°C)** |
| --- | --- | --- | --- |
| ACSL1  NM_012820.1 | TGTGGGGTGGAAATCATCGG  TTGGGGTTGCCTGTAGTTCC | 131 | 60 |
| CRAT  NM_001004085.2 | CTGCCAGAACCGTGGTGAAA  GGTGGGCCTTAAATCGACCA | 81 | 60 |
| CPT1A  NM_031559.2 | GTGCAGAGCAATAGGTCCCC  AGGCAGATCTGTTTGAGGGC | 125 | 60 |
| CPT2  NM_012930.1 | CTACATCTCAGGCCCCTGGT  GCCCTGGTAAGCTGGTCATT | 126 | 60 |
| ACADL  NM_012819.1 | TCCGCTTCCATGGCGAAATA  GCATCCACGTAGGCTTTTGC | 125 | 60 |
| ACADM  NM_016986.2 | GGCATATGGGTGTACAGGGG  ACGCAGTAGGCACACATCAT | 144 | 60 |
| ECH1  NM_022594.1 | GATGCAGCAGCTGAGAGGTA  CTTCTCCGGCCGGTTTAGTT | 184 | 60 |
| HADH  NM_057186.2 | TCGTGAACCGTCTCTTGGTG  TCCTTAGATGCATCGCCTCG | 73 | 60 |
| ACAT1  NM_017075.2 | AGTAACAGCTGCTAACGCCA  AGCAAATGCTGCGATTCGTG | 118 | 60 |
| PCCA  NM_019330.1 | TTGGCAGCAGAAGATGTCACT  CACAGGGTAGCCAATTTCCCT | 183 | 60 |
| PCCB  NM_017030.2 | TCACCAGGAAGGCCTATGGA  AAGATGATCTCCACGGCACC | 130 | 60 |
| MCEE  NM_001106341.1 | CCTGCCCACTCTCCTAGTTG  GAGAAAAGCCCTGTAGCGCC | 117 | 58 |
| MMUT  XM_003754495.4 | CAGCACAGCACATGCCAAAA  CGCGATGGTATAGGCCAGTT | 101 | 60 |
| ECI1  NM_017306.4 | GGTTCACCATTCCGGACCAT  TCGCTGCTTGATCAGGTTGT | 83 | 60 |
| DECR1  NM_057197.2 | GTGTGATCGCGAGCAGGAAT  AGGATGCCCTGCAACTTTGA | 158 | 60 |
| PLA1A  NM_138882.1 | ATCGCCCATCAAAACCCACA  CAGACCGTCTTCTTGCTGTCA | 149 | 60 |
| PLA2G2A  NM_031598.3 | GCCATACCACCATCCCATCC  GACCTGAATTGAGCCAAAGGC | 92 | 56 |
| PLCG1  NM_013187.1 | CAGCAATCCTAGAGCCGGAG  TCAGGACGAATGGCGATCTG | 109 | 60 |
| PLD1  NM_030992.1 | TGTAGGATAGCCAATTTCCCCC  GTAAAAACAGAGGGGCTGGG | 131 | 60 |
| GDE1  NM_032615.2 | CCTGACGAAAGGATCCCCAC  AGAGCAGCAGAAGCCATATCT | 110 | 60 |
| PEMT  NM_013003.1 | CCCGCTGAGTTCATCACCAG  GAAGAGATCGGCCAGAACCA | 87 | 60 |
| PPARA  NM_013196.1 | GTCCTCTGGTTGTCCCCTTG  TCAGTCTTGGCTCGCCTCTA | 143 | 60 |
| NT5C2  XM_006223765.3 | CCTGGAGTGACCGCTTACAG  CAAACACCCGGTGATAGGCT | 99 | 60 |
| ADA  NM_130399.2 | GCGAGAGGCTGTGGACATAC  GCATGCGTCGTTTTGGGATT | 165 | 60 |
| PNP  NM_001106031.1 | TGACCGGGATATGAGGCAGA  AAGTAGACAGAGCTCTCCGC | 132 | 60 |
| XDH  NM_017154.1 | AGCTTTTCCAAGAGGTGCCA  ACGTGATTTTAGCATGCGCC | 183 | 60 |
| UOX  NM_053768.2 | CGACTATGGAAAGAATGATGAAGTG  CTCAGAGTCAACTGCACCGA | 138 | 60 |
| GSR  NM_053906.2 | TCCAGATGTCGATTGCCTGC  GATCGCAACTGGGGTGAGAA | 193 | 60 |
| GPX1  NM_030826.4 | GGACATCAGGAGAATGGCAAGA  GGCATTCCGCAGGAAGGTAA | 153 | 60 |
| GGT1  NM_053840.2 | TACAGAGGTCACACCCGACT  TCCCCGCCTTTTCTGGAATC | 96 | 64 |
| SMS  NM_001033899.1 | CATCTTTCAGGAGCAGGGGAT  GGCAAAGCTGCCATTCTTGTTC | 94 | 60 |
| CAT  NM_012520.2 | GTGCATGCATGACAACCAGG  GAATGTCCGCACCTGAGTGA | 163 | 60 |
| SOD1  NM_017050.1 | AAGAGAGGCATGTTGGAGACC  CGGCCAATGATGGAATGCTC | 115 | 60 |
| SOD2  NM_017051.2 | ACGCGACCTACGTGAACAAT  TAACATCTCCCTTGGCCAGC | 72 | 60 |
| Clock  NM_021856.2 | AAGATGACACAGCGGAGGTC  ACTGTGACATGCCTTGTGGG | 127 | 60 |
| Bmal1  NM_024362.2 | TGCCACTGACTACCAAGAAAGT  ATTTTGTCCCGACGCCTCTT | 138 | 60 |
| Per1  NM_001034125.1 | CTCTCCGCAACCAGGATACC  GCTAGGAGCTCTGAGAAGCG | 139 | 60 |
| Per2  NM_031678.1 | AAGTGACGGGTCGAGCAAAG  CATGTCGGGCTCTGGAATGA | 71 | 60 |
| Per3  NM_023978.2 | CCACCCTCTCCAGGTCATGT  CGCCACTGAAACCAAAACCAA | 125 | 60 |
| Cry1  NM_198750.2 | CCCACTAAAGCAAGGAAGAAGC  CCCGCATGCTTTCGTATCAGTT | 134 | 60 |
| Cry2  NM_133405.2 | GGACTACATCCGGCGATACC  GCCAATGATGCACTTAGCGG | 112 | 60 |
| Npas2  NM_001108214.2 | TCTTCTGAGAGGCAGCTTGAA  CAGGAGGGGCTAGGCACATT | 85 | 60 |
| Rev-ErbA  NM_001113422.1 | ATCTCGGTTGCCTCAGCATC  CTAGGCACCGAGCAGTAAGG | 78 | 60 |
| Mettl3  NM_001024794.1 | ATGTGCAGCCCAACTGGATT  CTGTGCTTAAACCGGGCAAC | 88 | 60 |
| CSNK1D  NM_139060.3 | CACCTCACAGATTCCCGGTC  AAGGAGAGTTCTCATCGGTGC | 72 | 60 |
| CSNK1E  NM_031617.1 | CTCTGCAAAGGCTACCCCTC  CTCTGCAAAGGCTACCCCTC | 125 | 60 |
| FBXL3  NM_001100568.1 | AGAGAAAGGAAGCTTTCCGCCC  TTCTCAGCAGTGCCTTCCTCA | 102 | 60 |

**Supplementary Table S2.** Saline female vs Dex female raw metabolite data (significant results)

| **Name** | **Molecular Weight** | **RT [min]** | **Ratio: (DF) / (SF)** | **P-value: (DF) / (SF)** |
| --- | --- | --- | --- | --- |
| 2,3,4,5-Tetrahydroxypentanal | 150.05415 | 1.023 | 6.228 | 1.3135E-07 |
| 3,16,23-Trihydroxy-12,29-dioxooleanan-21-yl 2-(methylamino)benzoate | 637.39498 | 10.892 | 0.278 | 8.9726E-06 |
|  | 609.36384 | 10.503 | 0.404 | 2.1542E-05 |
| 1,2-DILINOLEOYL-SN-GLYCERO-3-PHOSPHATIDYLETHANOLAMINE | 739.515 | 12.616 | 0.006 | 6.1809E-05 |
| Spermidine | 145.15781 | 0.974 | 1.869 | 8.4361E-05 |
|  | 649.43133 | 10.972 | 0.374 | 9.8836E-05 |
|  | 128.13133 | 0.974 | 1.827 | 0.0001056 |
| Ophthalmic acid | 289.12773 | 1.004 | 4.303 | 0.00035531 |
| O-(Hydroxy{(2R)-3-(tetradecanoyloxy)-2-[(9Z)-9-tetradecenoyloxy]propoxy}phosphoryl)-L-serine | 677.42625 | 10.97 | 0.491 | 0.00121946 |
| Creatinine | 113.05918 | 1.009 | 1.905 | 0.00122754 |
| N-[(2S,3R,4E)-1-{[4-O-(beta-D-Galactopyranosyl)-beta-D-glucopyranosyl]oxy}-3-hydroxy-4-octadecen-2-yl]dodecanamide | 805.54891 | 12.409 | 0.216 | 0.00175637 |
|  | 244.15345 | 1.174 | 1.805 | 0.0022134 |
| Benserazide | 257.10248 | 1.008 | 2.048 | 0.00288048 |
| (2S,4R,4aS,4bR,6aS,8aR,11aR,15bS,15cS,17aS)-4-Hydroxy-2-(2-hydroxy-2-propanyl)-9,9,11,11,15b,15c-hexamethyl-12-oxo-3,4,5,6,6a,7,8,8a,9,11,11a,12,15,15b,15c,16,17,17a-octadecahydro-2H-[2]benzofuro[5,6- e]chromeno[5',6':6,7]indeno[1,2-b]indole-4a(4bH)-carbaldehyde | 617.36883 | 10.611 | 0.463 | 0.00292188 |
|  | 621.39993 | 11.107 | 0.5 | 0.00338076 |
|  | 579.35326 | 10.733 | 0.576 | 0.00433824 |
| Adenosine | 267.09668 | 2.669 | 1.97 | 0.00473755 |
|  | 593.36885 | 10.751 | 0.539 | 0.00502002 |
| Adenine | 135.05404 | 2.672 | 1.911 | 0.0109412 |
| Elaidolinolenic acid | 278.22449 | 9.748 | 0.308 | 0.01164811 |
| Pregabalin | 159.12588 | 1.002 | 1.723 | 0.01502947 |
|  | 313.10272 | 2.674 | 2.081 | 0.01516026 |
| 3-Hydroxy-3-[(3-methylbutanoyl)oxy]-4-(trimethylammonio)butanoate | 261.1575 | 1.251 | 1.486 | 0.0186267 |
| Choline | 103.10006 | 0.951 | 1.683 | 0.02210976 |
| Propionylcarnitine | 217.13125 | 1.71 | 2.059 | 0.02685635 |
|  | 175.12076 | 1.011 | 1.579 | 0.03285393 |
|  | 635.3796 | 10.615 | 0.581 | 0.03286697 |
| Hypoxanthine | 136.03851 | 1.328 | 1.634 | 0.03538884 |
| L-(+)-ERGOTHIONEINE | 229.08838 | 0.976 | 1.558 | 0.03850724 |
| Glutathione disulfide | 612.15218 | 1.021 | 1.775 | 0.04162925 |
| Nicotinamide | 122.04825 | 1.599 | 1.464 | 0.04325364 |
| 5-Nitro-o-toluidine | 152.05852 | 1.304 | 1.449 | 0.0448727 |
| Benzoxazolone | 135.03216 | 1.3 | 1.415 | 0.0460894 |
|  | 829.54576 | 11.411 | 0.417 | 0.04699072 |
| 7-Chloro-5-(2-fluorophenyl)-1-(2-methoxyethyl)-1,3-dihydro-2H-1,4-benzodiazepin-2-one | 346.08638 | 2.492 | 1.525 | 0.0472046 |
|  | 574.11782 | 1.319 | 1.621 | 0.04741469 |
| Caprolactam | 113.08438 | 4.698 | 1.394 | 0.04861032 |
| L-(-)-methionine | 149.05102 | 1.029 | 1.667 | 0.04927301 |

**Supplementary Table S3.** Saline male vs Dex male raw metabolite data of significant results

| **Name** | **Molecular Weight** | **RT [min]** | **Ratio:**  **(DM) / (SM)** | **P-value (DM) / (SM)** |
| --- | --- | --- | --- | --- |
| 1-[(9Z)-octadecenoyl]-2-[(4Z,7Z,10Z,13Z,16Z,19Z)-docosahexaenoyl]-sn-glycero-3-phosphocholine | 831.57634 | 12.968 | 0.265 | 0.00054184 |
| 4-Formyl-2-methoxyphenyl hydrogen sulfate | 232.00407 | 5.906 | 3.004 | 0.00351615 |
|  | 649.43133 | 10.972 | 0.445 | 0.00708704 |
|  | 782.56389 | 13.032 | 0.307 | 0.00823096 |
|  | 808.57807 | 12.886 | 8.995 | 0.00883183 |
| DL-Carnitine | 161.10507 | 0.752 | 0.487 | 0.01061418 |
| DL-TYROSINE | 181.0736 | 1.068 | 0.63 | 0.01128288 |
| Xanthine | 152.03346 | 1.115 | 0.623 | 0.0124001 |
|  | 474.29015 | 10.685 | 0.602 | 0.02156628 |
| (E)-p-coumaric acid | 164.04733 | 1.064 | 0.68 | 0.02293774 |
|  | 565.31405 | 10.595 | 0.247 | 0.0234832 |
| Dihexyverine | 321.26642 | 10.613 | 0.306 | 0.02482968 |
| DL-Tryptophan | 204.08954 | 2.705 | 0.77 | 0.02914266 |
| 4149853 | 371.3033 | 10.799 | 0.203 | 0.03287597 |
| 9,10-Dimethoxy-1,2,3,6,7,12,15,16-octadehydrogalanthan-6-ium | 266.11997 | 6.04 | 0.55 | 0.03435221 |
| Xanthine | 152.03274 | 1.088 | 0.634 | 0.0356631 |
|  | 600.3371 | 10.947 | 0.241 | 0.03704887 |
|  | 809.58238 | 13.189 | 0.228 | 0.03816212 |
| SL3675000 | 262.22936 | 10.778 | 0.186 | 0.03849424 |
| Uracil | 112.02624 | 1.028 | 0.464 | 0.04046308 |
| Hypoxanthine | 136.03828 | 1.059 | 0.717 | 0.0405606 |
| LysoPC(18:3(9Z,12Z,15Z)) | 517.31415 | 10.781 | 0.355 | 0.04166692 |
|  | 442.18924 | 7.337 | 2.307 | 0.042561 |
| 3,16,23-Trihydroxy-12,29-dioxooleanan-21-yl 2-(methylamino)benzoate | 637.39498 | 10.892 | 0.565 | 0.04479033 |
| Alpha.-Aminoadipic acid | 161.06873 | 0.791 | 0.212 | 0.0472026 |
| Eicosapentanoic acid | 302.22421 | 10.569 | 0.43 | 0.04859053 |

**Supplementary Table S4.** Female pathway analysis results generated through the MetaboAnalyst 4.0 pathway analysis tool. Match status indicates the number of metabolites highlighted in the screen/the total number of metabolites in the pathway.

| **Pathway Name** | **Match Status** | **p** |
| --- | --- | --- |
| Purine metabolism | 3/66 | 0.020585 |
| Glutathione metabolism | 2/28 | 0.026334 |
| Glycerophospholipid metabolism | 2/36 | 0.04207 |
| Glycosylphosphatidylinositol (GPI)-anchor biosynthesis | 1/14 | 0.12284 |
| Nicotinate and nicotinamide metabolism | 1/15 | 0.13105 |
| Pentose and glucuronate interconversions | 1/18 | 0.15527 |
| Beta-Alanine metabolism | 1/21 | 0.17886 |
| Sphingolipid metabolism | 1/21 | 0.17886 |
| Cysteine and methionine metabolism | 1/33 | 0.26723 |
| Glycine, serine and threonine metabolism | 1/34 | 0.27418 |
| Arginine and proline metabolism | 1/38 | 0.30137 |
| Aminoacyl-tRNA biosynthesis | 1/48 | 0.36527 |

**Supplementary Table S5.** Male pathway analysis results generated through the MetaboAnalyst 4.0 pathway analysis tool. Match status indicates the number of metabolites highlighted in the screen/the total number of metabolites in the pathway.

| **Pathway Name** | **Match Status** | **p** |
| --- | --- | --- |
| Glycerophospholipid metabolism | 2/36 | 0.022085 |
| Phenylalanine, tyrosine and tryptophan biosynthesis | 1/4 | 0.026271 |
| Linoleic acid metabolism | 1/5 | 0.032741 |
| Aminoacyl-tRNA biosynthesis | 2/48 | 0.037894 |
| Ubiquinone and other terpenoid-quinone biosynthesis | 1/9 | 0.058236 |
| Purine metabolism | 2/66 | 0.067597 |
| Phenylalanine metabolism | 1/12 | 0.076958 |
| Alpha-Linolenic acid metabolism | 1/13 | 0.083124 |
| Pantothenate and CoA biosynthesis | 1/19 | 0.11935 |
| Beta-Alanine metabolism | 1/21 | 0.13113 |
| Lysine degradation | 1/25 | 0.15428 |
| Arachidonic acid metabolism | 1/36 | 0.21509 |
| Biosynthesis of unsaturated fatty acids | 1/36 | 0.21509 |
| Pyrimidine metabolism | 1/39 | 0.23098 |
| Tryptophan metabolism | 1/41 | 0.24141 |
| Tyrosine metabolism | 1/42 | 0.24658 |
